# Composite Fe_3_O_4_-MXene-Carbon Nanotube Electrodes for Supercapacitors Prepared Using the New Colloidal Method

**DOI:** 10.3390/ma14112930

**Published:** 2021-05-29

**Authors:** Wenyu Liang, Igor Zhitomirsky

**Affiliations:** Department of Materials Science and Engineering, McMaster University, Hamilton, ON L8S 4L7, Canada; liangw26@mcmaster.ca

**Keywords:** iron oxide, MXene, supercapacitor, electrode, dispersion, composite, carbon nanotube

## Abstract

MXenes, such as Ti_3_C_2_T_x_, are promising materials for electrodes of supercapacitors (SCs). Colloidal techniques have potential for the fabrication of advanced Ti_3_C_2_T_x_ composites with high areal capacitance (C_S_). This paper reports the fabrication of Ti_3_C_2_T_X_-Fe_3_O_4_-multiwalled carbon nanotube (CNT) electrodes, which show C_S_ of 5.52 F cm^−2^ in the negative potential range in 0.5 M Na_2_SO_4_ electrolyte. Good capacitive performance is achieved at a mass loading of 35 mg cm^−2^ due to the use of Celestine blue (CB) as a co-dispersant for individual materials. The mechanisms of CB adsorption on Ti_3_C_2_T_X_, Fe_3_O_4_, and CNTs and their electrostatic co-dispersion are discussed. The comparison of the capacitive behavior of Ti_3_C_2_T_X_-Fe_3_O_4_-CNT electrodes with Ti_3_C_2_T_X_-CNT and Fe_3_O_4_-CNT electrodes for the same active mass, electrode thickness and CNT content reveals a synergistic effect of the individual capacitive materials, which is observed due to the use of CB. The high C_S_ of Ti_3_C_2_T_X_-Fe_3_O_4_-CNT composites makes them promising materials for application in negative electrodes of asymmetric SC devices.

## 1. Introduction

Ti_3_C_2_T_x_ belongs to the family of MXene-type materials, which are of great technological interest for applications in electrodes of SCs [1,2,3]. The interest in Ti_3_C_2_T_x_ is attributed to the high capacitance and low electrical resistivity of this material. The promising capacitive properties of Ti_3_C_2_T_x_ result from its high surface area and the redox active nature of surface functional groups. Enhanced capacitive properties were obtained for Ti_3_C_2_T_x_ composites, containing different conductive additives, such as graphene [4], acetylene black [5], and carbon black [6] and for nitrogen-doped Ti_3_C_2_T_x_ [7,8,9]. Moreover, advanced Ti_3_C_2_T_x_ composites were developed, containing other components, such as ZnO [10], MnO_2_ [11], TiO_2_ [12], and Mn_3_O_4_ [13]. Investigations revealed the stable cycling behavior of Ti_3_C_2_T_x_ composites [14,15,16,17,18,19].

High specific capacitance (C_m_) normalized by active mass (AM) was reported for composite electrodes [9,12,14,20,21,22,23,24,25,26,27,28] with relatively low AMs, typically below 8 mg∙cm^−2^. The C_S_ of such electrodes was below 1 F∙cm^−2^. Capacitive properties of Ti_3_C_2_T_x_ composites were tested in various electrolytes, such as HCl [29], H_2_SO_4_ [27,30,31], KOH [12,32], KCl [33], K_2_SO_4_ [34], Na_2_SO_4_ [34], Li_2_SO_4_ [34], and other electrolytes [35,36]. Ti_3_C_2_T_x_-based electrodes were utilized for the fabrication of symmetric SCs, containing two similar Ti_3_C_2_T_x_-based electrodes, with maximum operation voltages in the range of 0.4–1.2 V [9,31,37,38].

The progress in applications of SC devices will depend on the ability to fabricate efficient electrodes and devices with high C_S_, which can be achieved at high AM loadings. Another important benefit of high AM electrodes is their low ratio of the mass of electrochemically inactive components to the AMs. With the goal to increase energy–power characteristics, there is a growing trend in devices that operate in enlarged voltage windows. Of particular importance are environmentally friendly neutral electrolytes, such as Na_2_SO_4_, which facilitate the design of asymmetric aqueous cells with voltage windows above 1.2 V.

Ti_3_C_2_T_x_-based electrodes with AMs of 1–3 mg cm^−2^ were analyzed in Na_2_SO_4_ electrolyte [34,39,40] and relatively high C_m_ were obtained at such low AM loadings. Therefore, the development of electrodes with higher AMs can potentially result in high C_S_. However, it is challenging [41] to achieve high C_S_ owing to the electrolyte diffusion limitations and high electrical resistance at high AMs. The increase in AM to the level of 20 mg∙cm^−2^ allowed the design of composites [42] with C_S_ of 1.087 F∙cm^−2^ at the galvanostatic charging conditions of 1 mA∙cm^−2^ and 0.783 F∙cm^−2^ at potential sweep conditions of 1 mV∙s^−1^. Such electrodes [42] were utilized for symmetric Ti_3_C_2_T_x_ SC.

The objective of this study was to form Fe_3_O_4_-Ti_3_C_2_T_x_-CNT electrodes for SCs. The use of CB as a co-dispersant allowed the fabrication of electrodes, which showed good electrochemical performance at AM of 35 mg∙cm^−^^2^. CB allowed adsorption on individual materials and their dispersion due its polyaromatic structure, containing a chelating catechol ligand and electric charge. The experimental data of this investigation showed that C_S_ of 5.52 F∙cm^−2^ can be achieved in the negative potential range in 0.5 M Na_2_SO_4_ electrolyte due to the use of advanced co-dispersant and a synergistic effect of the individual components.

## 2. Materials and Methods

Celestine blue (CB), FeCl_3_·6H_2_O, FeCl_2_·4H_2_O, NH_4_OH, Na_2_SO_4_, co-polymer of vinyl butyral, vinyl acetate and vinyl alcohol (PVBAA, 65 kDa) were purchased from Millipore Sigma, Burlington, MA, USA. The diameter and length of CNT (multiwalled, Bayer Corp. Whippany, NJ, USA) were 13 nm and 1–2 μm, respectively. Ti_3_C_2_T_x_ was purchased from Laizhou Kai Kai Ceramic Materials Co., Ltd., Laizhou, China. Fe_3_O_4_ was prepared as described in by a chemical precipitation method [43] from solutions of FeCl_2_ and FeCl_3_, containing dispersed CNT or co-dispersed CNT and Ti_3_C_2_T_x_. In contrast to the previous investigation [43], pristine CNT were used. In this approach, CNT and Ti_3_C_2_T_x_ were dispersed or co-dispersed using CB as a surfactant. For the fabrication of Fe_3_O_4_-CNT electrodes, the synthesis of Fe_3_O_4_ was performed in the presence of CNT, dispersed using CB. For the fabrication of Ti_3_C_2_T_x_-CNT electrodes, Ti_3_C_2_T_x_ was co-dispersed with CNT in water using CB as a co-dispersant. Active materials (AM) for Ti_3_C_2_T_x_-Fe_3_O_4_-CNT electrodes were prepared by precipitating Fe_3_O_4_ in the presence of co-dispersed Ti_3_C_2_T_x_ and CNT. The amount of the CB dispersant in the suspension was 15% of the total mass of Ti_3_C_2_T_x_, Fe_3_O_4_ and CNT. After filtration, obtained AM were washed with water and ethanol in order to remove non-adsorbed dispersant and dried in air. In order to analyze the effect of CB, AM for Ti_3_C_2_T_x_-(Fe_3_O_4_-CNT) electrodes were prepared by fabrication of Fe_3_O_4_-CNT powder, as described above, and its mixing with Ti_3_C_2_T_x_. The Ti_3_C_2_T_x_/Fe_3_O_4_ mass ratio was 5:3 in the Ti_3_C_2_T_x_-(Fe_3_O_4_-CNT) and Ti_3_C_2_T_x_-Fe_3_O_4_-CNT electrodes. The mass ratio of CNT to the mass of active materials, such as Fe_3_O_4_ in Fe_3_O_4_-CNT, Ti_3_C_2_T_x_ in Ti_3_C_2_T_x_-CNT, Fe_3_O_4_ and Ti_3_C_2_T_x_ (total) in Ti_3_C_2_T_x_-(Fe_3_O_4_-CNT) and Ti_3_C_2_T_x_-Fe_3_O_4_-CNT was 1:4.

Obtained powders were used for the fabrication of slurries in ethanol for the impregnation of commercial Ni foam current collectors (95% porosity, Vale, Rio de Janeiro, Brazil). The slurries contained dissolved PVBAA binder. The mass of the binder was 3% of the total mass of the active material (AM). The total AM of impregnated material after drying was 35 mg cm^−2^, which included 3% PVBAA binder. All of the impregnated Ni foams were pressed using a calendering machine in order to obtain a final electrode thickness of 0.38 mm.

Electrochemical impedance spectroscopy (EIS) and cyclic voltammetry (CV) studies were performed using a potentiostat (PARSTAT 2273, AMETEK, Berwyn, PA, USA). Galvanostatic charge discharge (GCD) was conducted using a Biologic AMP 300 potentiostat. The capacitive behavior of the electrodes was tested in an aqueous 0.5 M Na_2_SO_4_ solution. Pt gauze was utilized as a counter electrode, and a saturated calomel electrode (SCE) was used as a reference. The area of the working electrode was 1 cm^2^. Capacitances C_S_ and C_m,_ normalized by the electrode area or mass of the active material, respectively, were obtained from the CV or GCD data, and complex C_S_* components (C_S_’ and C_S_”) were calculated from the EIS testing results obtained at a signal of 5 mV, as described in [41]. JSM-7000F microscope (JEOL, Peabody, MA, USA) was used for SEM investigations.

## 3. Results and Discussion

**Figure 1:**
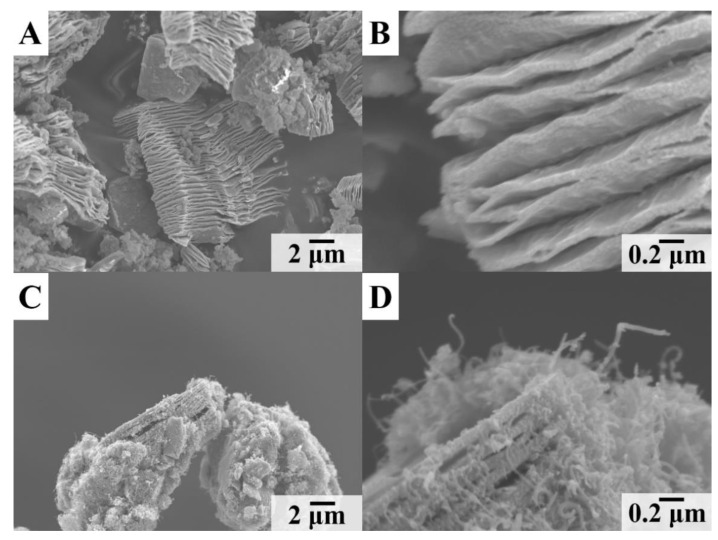
SEM images at different magnifications of (**A**,**B**) as-received Ti_3_C_2_T_x_ and (**C**,**D**) Ti_3_C_2_T_X_-Fe_3_O_4_-CNT.

Figure 1A,B shows SEM images of Ti_3_C_2_T_x_ particles used in this investigation. The particles exhibit an accordion-like structure, which is beneficial for electrolyte access to the material. However, some small pores may not be accessible by the electrolyte. It is in this regard that the investigations of other pseudocapacitive materials did not show correlation between BET surface area and capacitance [44,45,46,47]. The SEM images of Ti_3_C_2_T_X_-Fe_3_O_4_-CNT composites (Figure 1C,D) show that Ti_3_C_2_T_X_ particles were covered with Fe_3_O_4_ and CNT.

Ti_3_C_2_T_x_ particles were used for the fabrication of composite Ti_3_C_2_T_x_-Fe_3_O_4_-CNT electrodes. Pure Ti_3_C_2_T_x_-CNT and Fe_3_O_4_-CNT electrodes were also fabricated and tested for comparison. The X-ray diffraction patterns of the composite Ti_3_C_2_T_x_-CNT, Fe_3_O_4_-CNT, and Ti_3_C_2_T_x_-Fe_3_O_4_-CNT materials presented in the Supplementary Information (Figure S1) show diffraction peaks of the individual components. All the electrodes contained 20% CNTs as conductive additives. In this investigation, CNTs were used as conductive additives for capacitive Fe_3_O_4_ [48,49,50] and Ti_3_C_2_T_x_ [1,2,3] materials. Previous investigations highlighted the need for the fabrication of electrodes with high AMs and enhanced ratio of the AM to the mass of current collector and other passive components [41]. Commonly used so far are activated carbon (AC) commercial supercapacitors with high AM [41,51] of about 10 mg∙cm^−2^. Another important parameter is electrode thickness [52]. It has been demonstrated that significant uncertainty in supercapacitor metrics stems from reporting gravimetric capacitance of thick electrodes with low packing density [51]. In such electrodes, empty space is filled by an electrolyte, thereby increasing the weight of the device without adding capacitance. However, such electrodes show enhanced AM normalized capacitance due to enhanced access of the electrolyte to the active materials [51]. Investigations showed that electrodes must be of comparable thickness for the comparison of their performance [53]. It is important to note that AC has a relatively low density and typical thickness of AC electrodes with active mass of 10 mg∙cm^−2^ is about 0.6 mm [54]. In our investigation, the thickness of all the investigated electrodes was 0.38 mm and AM loading was 35 mg∙cm^−2^. The higher AM of the fabricated electrodes, compared to that of AC electrodes, resulted from higher density of Ti_3_C_2_T_x_ and Fe_3_O_4_ materials used in this investigation. The high AM loading was beneficial for increasing the ratio of AM - to the total mass, which includes not only AM, but also mass of current collectors, electrolyte and other components.The ability to achieve high capacitance using electrodes with high AM and low impedance is critical for the development of advanced electrodes.

In this investigation, CB was used as a dispersant for Ti_3_C_2_T_x_, Fe_3_O_4_ and CNTs. CB has generated significant interest as an advanced dispersant for the fabrication of composites for supercapacitors and other applications [55,56,57]. Sedimentation tests showed good colloidal stability of the Ti_3_C_2_T_x_, Fe_3_O_4_ and CNT suspensions, prepared using CB. It is important to note that the chemical structure of CB contains a catechol ligand, which facilitates CB adsorption on inorganic materials by complexation of metal atoms on the material surface [58]. Such interactions of CB with Ti atoms on the Ti_3_C_2_T_x_ surface or Fe atoms on the Fe_3_O_4_ surface facilitated CB adsorption. The polyaromatic structure of CB allowed for its adsorption on CNTs and the adsorption mechanism of CB involved π-π interactions with side walls of CNTs [59]. The adsorbed cationic CB allowed for electrostatic dispersion of Ti_3_C_2_T_x_, Fe_3_O_4_ and CNT and facilitated their enhanced mixing. Co-dispersion of Ti_3_C_2_T_x_ with CNTs and Fe_3_O_4_ with CNTs allowed for good performance of Ti_3_C_2_T_x_-CNT and Fe_3_O_4_-CNT electrodes at high AM loadings.

**Figure 2:**
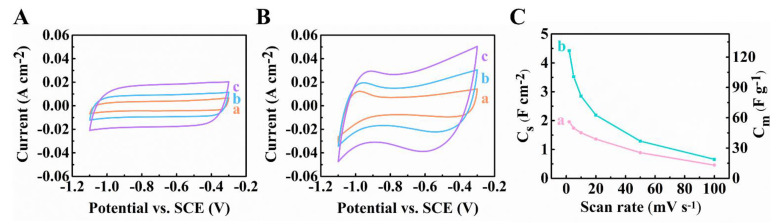
(**A**,**B**) Cyclic voltammetry data at (a) 2, (b) 5 and (c) 10 mV s^−1^, (**C**) capacitances for ((**A**,**C**) (a)) Ti_3_C_2_T_X_-CNT and ((**B**,**C**) (b)) Fe_3_O_4_-CNT electrodes.

Figure 2 shows capacitive performances of Ti_3_C_2_T_x_-CNT and Fe_3_O_4_-CNT electrodes. Cyclic voltammetry (CV) studies showed nearly rectangular shape CVs for Ti_3_C_2_T_x_-CNT electrodes and C_S_ = 1.96 F∙cm^−2^ at 2 mV s^−1^. The obtained C_S_ was significantly higher than literature data for Ti_3_C_2_T_x_ based electrodes, discussed in the Introduction. The capacitance retention at 100 mV∙s^−1^ was 23.5%. Relatively high capacitances were also achieved using Fe_3_O_4_-CNT electrodes. The highest C_S_ = 4.42 F∙cm^−2^ was attained at 2 mV∙s^−1^. The use of CB as a co-dispersant allowed for higher capacitance of the Fe_3_O_4_-CNT electrodes compared to the previous results [43] for the Fe_3_O_4_-CNT electrodes, containing functionalized CNTs. The capacitance retention at 100 mV s^−1^ was 14.9%. The capacitive properties of Fe_3_O_4_-CNT composites resulted from the double layer charging mechanism of Fe_3_O_4_ and CNTs and pseudocapacitive mechanism of Fe_3_O_4_, attributed to Fe^2+^/Fe^3+^ redox couple [48,49,50].

**Figure 3:**
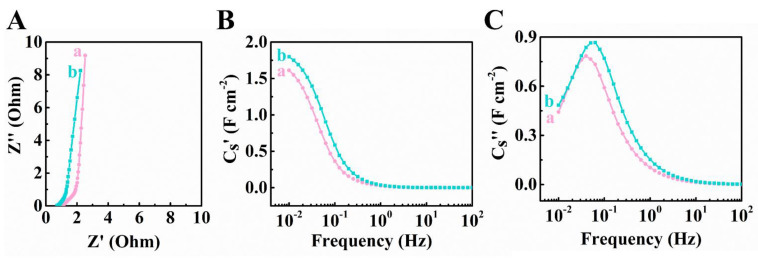
(**A**) Nyquist Z” vs. Z’ graph for EIS data, (**B**) C_S_’ and (**C**) C_S_”, derived from the EIS data for (a) Ti_3_C_2_T_X_-CNT and (b) Fe_3_O_4_-CNT electrodes.

Figure 3 shows EIS data for the Ti_3_C_2_T_x_-CNT and Fe_3_O_4_-CNT electrodes. The Nyquist plot of complex impedance revealed lower resistance, R = Z’, compared to the literature data [42]. The low electrical resistance is an important factor controlling capacitive performance of electrodes. The differential capacitance C_S_’ derived from the EIS data at 5 mV signal amplitude was inferior to the integral C_S_ calculated for potential span of 0.8 V. The discrepancy can be attributed to different parameters, such as charge–discharge time, electrode potential and limited accessibility of some redox sites at low voltages. The electrodes showed relatively high relaxation frequencies [60,61], corresponding to C_S_” maxima.

**Figure 4:**
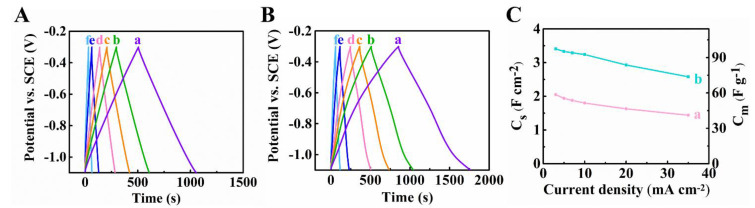
Galvanostatic charge–discharge curves of (**A**)Ti_3_C_2_T_X_-CNT, (**B**) Fe_3_O_4_-CNT at (a) 3, (b) 5 (c) 7, (d) 10 (e) 20 and (f) 35 mA∙cm^−2^, (**C**) capacitances derived from GCD tests for (a) Ti_3_C_2_T_X_-CNT and (b) Fe_3_O_4_-CNT electrodes.

Figure 4A,B shows charge-discharge behavior of the Ti_3_C_2_T_X_-CNT and Fe_3_O_4_-CNT electrodes. The electrodes showed nearly triangular symmetric GCD profile. The capacitances were calculated from the GCD data and are presented in Figure 4C. C_S_ reduced from 2.05 to 1.40 F∙cm^−2^ and from 3.41 to 2.5 F∙cm^−2^, for Ti_3_C_2_T_X_-CNT and Fe_3_O_4_-CNT electrodes, respectively, in the current range 3–35 mA∙cm^−2^. The GCD data showed good capacitance retention with increasing current density.

This investigation revealed a synergistic effect of Ti_3_C_2_T_X_, CNT and Fe_3_O_4_, which allowed for enhanced capacitance of the composite Ti_3_C_2_T_X_-Fe_3_O_4_-CNT electrodes, compared to the capacitances of Ti_3_C_2_T_X_-CNT and Fe_3_O_4_-CNT electrodes at the same AM, electrode thickness and CNT content. The use of CB as a dispersant was critical to achieve enhanced capacitance. The effect of CB is evident from the comparison of testing results for two composites, prepared at different experimental conditions, as was described in the Materials and Methods section. Ti_3_C_2_T_X_-(Fe_3_O_4_-CNT) electrodes were prepared by precipitation of Fe_3_O_4_ in the presence of CNTs dispersed with CB, followed by washing drying and mixing with Ti_3_C_2_T_X_. In contrast Ti_3_C_2_T_X_-Fe_3_O_4_-CNT electrodes were prepared by precipitation of Fe_3_O_4_ in the presence of co-dispersed Ti_3_C_2_T_X_ and CNTs.

**Figure 5:**
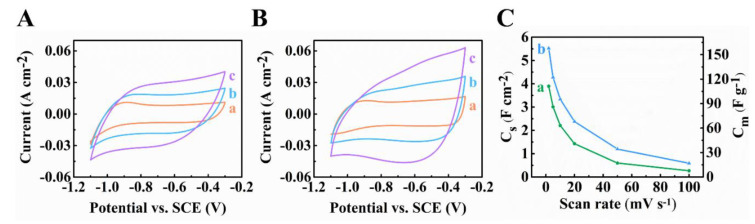
(**A**,**B**) Cyclic voltammetry data at (a) 2, (b) 5 and (c) 10 mV∙s^−1^, (**C**) capacitances for ((**A**,**C**) (a)) Ti_3_C_2_T_X_-(Fe_3_O_4_-CNT) and ((**B**,**C**) (b)) Ti_3_C_2_T_X_-Fe_3_O_4_-CNT electrodes.

CV testing results showed significantly larger CV areas for Ti_3_C_2_T_X_-Fe_3_O_4_-CNT, compared to Ti_3_C_2_T_X_-(Fe_3_O_4_-CNT) electrodes (Figure 5A,B). This resulted in higher capacitance of the Ti_3_C_2_T_X_-Fe_3_O_4_-CNT and indicated the influence of CB dispersant used for the preparation of the composites on the properties of the electrodes. The highest capacitances of 5.52 and 3.90 F∙cm^−2^ were obtained for Ti_3_C_2_T_X_-Fe_3_O_4_-CNT and Ti_3_C_2_T_X_-(Fe_3_O_4_-CNT) electrodes, respectively, at 2 mV∙s^−1^. In order to analyze the charge storage properties of the electrodes, a parameter b was calculated from the following equation [62,63].

*i* = aν^b^ (1)

where *i* is a current, ν—scan rate and a is a parameter. Parameter b was found to be 0.68 for the Ti_3_C_2_T_X_-Fe_3_O_4_-CNT electrodes (Supplementary Information, Figure S2). It is known that b = 1 for purely double-layer capacitive mechanism and b = 0.5 for battery-type materials. The electrodes with 0.5 < b < 1 combine capacitive and battery properties. According to [62], the battery-type charge storage mechanism is dominant for electrodes with 0.5 < b < 0.8. Therefore, the Ti_3_C_2_T_X_-Fe_3_O_4_-CNT electrodes show mixed double-layer capacitive and battery-type properties with a dominant battery-type charge storage mechanism.

**Figure 6:**
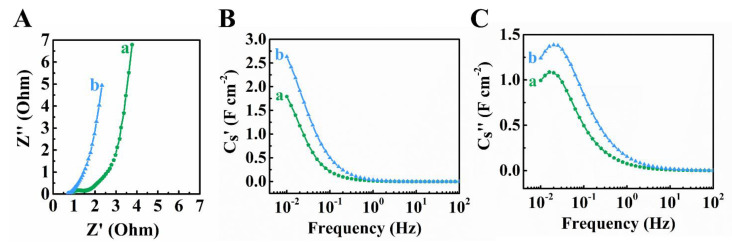
(**A**) Nyquist Z” vs. Z’ graph for EIS data, ((**B**,**C**)), (**B**) C_S_’ and (**C**) C_S_”, derived from the EIS data for (a) Ti_3_C_2_T_X_- (Fe_3_O_4_-CNT) and (b) Ti_3_C_2_T_X_-Fe_3_O_4_-CNT electrodes.

**Figure 7:**
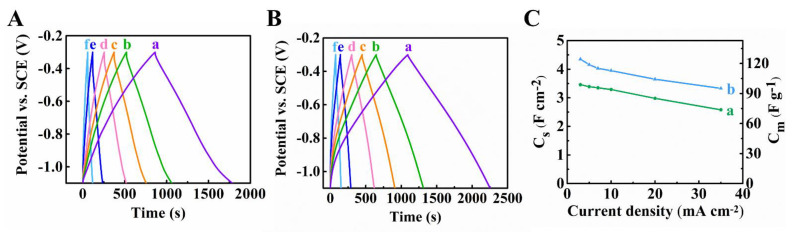
GCD curves for (**A**) Ti_3_C_2_T_X_-Fe_3_O_4_-CNT), (**B**) Ti_3_C_2_T_X_-Fe_3_O_4_-CNT at (a) 3, (b) 5 (c) 7, (d) 10 (e) 20 and (f) 35 mA∙cm^−2^, (**C**) capacitances versus current density, calculated from GCD data for (a) Ti_3_C_2_T_X_-(Fe_3_O_4_-CNT) and (b) Ti_3_C_2_T_X_-Fe_3_O_4_-CNT.

EIS studies (Figure 6) revealed lower resistance, higher capacitance and higher relaxation frequency of Ti_3_C_2_T_X_-Fe_3_O_4_-CNT electrodes, compared to Ti_3_C_2_T_X_-(Fe_3_O_4_-CNT) electrodes. GCD data showed nearly triangular symmetric charge–discharge curves, with longer charge and discharge times for Ti_3_C_2_T_X_-Fe_3_O_4_-CNT electrodes, compared to Ti_3_C_2_T_X_-(Fe_3_O_4_-CNT) at the same current densities (Figure 7A,B). The longer charge/discharge times indicated higher capacitances. The capacitances were calculated from the GCD data and presented in Figure 7C at different current densities. C_S_ reduced from 4.35 to 3.33 F∙cm^−2^ and from 3.46 to 2.58 F∙cm^−2^ for Ti_3_C_2_T_X_-Fe_3_O_4_-CNT and Ti_3_C_2_T_X_-(Fe_3_O_4_-CNT) composites, respectively, with current increase from 3 to 35 mA∙cm^−2^.

**Figure 8:**
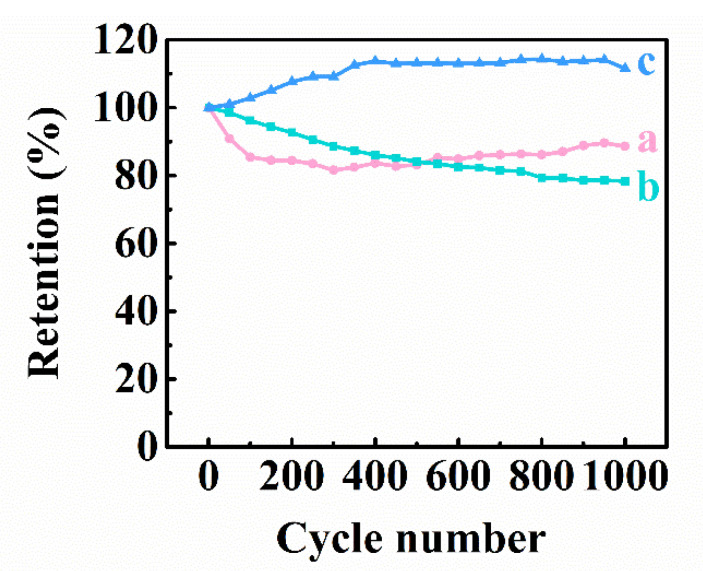
Capacitance retention for (a) Ti_3_C_2_T_X_-CNT, (b) Fe_3_O_4_-CNT and (c) Ti_3_C_2_T_X_-Fe_3_O_4_-CNT electrodes.

The analysis of capacitances, measured using CV, EIS and GCD techniques showed that the capacitances of the Ti_3_C_2_T_X_-Fe_3_O_4_-CNT electrodes are higher than the capacitances of the Ti_3_C_2_T_x_-CNT and Fe_3_O_4_-CNT electrodes. Therefore, the experimental results of this work showed a synergistic effect of the individual capacitive materials. The comparison of the data for Ti_3_C_2_T_X_-Fe_3_O_4_-CNT and Ti_3_C_2_T_X_-(Fe_3_O_4_-CNT) electrodes and literature data of the previous investigations for Ti_3_C_2_T_X_ [42] and Fe_3_O_4_ electrodes [43] showed the beneficial effect of co-dispersion of the individual components, which was achieved using CB as a dispersant. The ability to achieve high C_S_ of 5.52 F∙cm^−2^ in the negative potential range in Na_2_SO_4_ is beneficial for the preparation of asymmetric SC. Ti_3_C_2_T_X_-Fe_3_O_4_-CNT electrodes showed relatively high C_S_, compared to other anode materials [41]. The comparison with C_S_ for other Ti_3_C_2_T_X_-based electrodes in Na_2_SO_4_ electrolyte (Supplementary Information, Table S1) showed significant improvement in C_S_. The capacitance of the negative electrodes is usually lower than that of positive electrodes. Advanced positive electrodes, based on MnO_2_, Mn_3_O_4_, and BiMn_2_O_5_ have been developed with capacitance of about 5–8 F cm^−2^ in the positive potential range [41]. Therefore, the capacitance of Ti_3_C_2_T_X_-Fe_3_O_4_-CNTs is comparable with capacitances of advanced positive electrodes. The Ti_3_C_2_T_X_-Fe_3_O_4_-CNT electrodes showed a slight C_S_ increase for the first 400 cycles and remained nearly constant after this initial increase (Figure 8). A similar increase was observed in the literature for other materials and was attributed to microstructure changes during initial cycling [64,65]. In contrast, the capacitance of the Ti_3_C_2_T_X_- CNT and Fe_3_O_4_-CNT electrodes decreased after cycling (Figure 8).

## 4. Conclusions

Ti_3_C_2_T_X_-Fe_3_O_4_-CNT electrodes have been developed, which showed C_S_ of 5.52 F∙cm^−2^ in the negative potential range in 0.5 M Na_2_SO_4_ electrolyte. Such electrodes are promising for applications in asymmetric supercapacitor devices due to the high capacitance, which is comparable with the capacitance of advanced positive electrodes. The use of CB as an advanced co-dispersant allowed for the fabrication of Ti_3_C_2_T_X_-Fe_3_O_4_-CNT electrodes, which showed good capacitive performance at high AM loadings. The comparison of capacitive behavior of Ti_3_C_2_T_X_-Fe_3_O_4_-CNT electrodes with Ti_3_C_2_T_X_-CNT and Fe_3_O_4_-CNT electrodes with the same AM, thickness and CNT content revealed a synergistic effect of the individual capacitive materials.

## Data Availability

The data presented in this study are available in: “Composite Fe_3_O_4_-MXene-carbon nanotube electrodes for supercapacitors prepared by new colloidal method”.

## Supplementary Materials

The following are available online at https://www.mdpi.com/article/10.3390/ma14112930/s1. Figure S1: X-ray diffraction patterns of (a) Ti_3_C_2_T_X_-CNT and (b) Fe_3_O_4_-CNT and (c) Ti_3_C_2_T_X_-Fe_3_O_4_-CNT composites, Figure S2: Current (i) versus scan rate (ν) dependence in a logarithmic scale used for the calculation of parameter b for Ti_3_C_2_T_X_-Fe_3_O_4_-CNT electrodes from the equation [1] *i* = aν^b^, Table S1: Characteristics of Ti_3_C_2_T_x_-based electrodes with high active mass in Na_2_SO_4_ electrolyte.
