# Supplementary material for: Composite Fe3O4-MXene-Carbon Nanotube Electrodes for Supercapacitors Prepared Using the New Colloidal Method
Source: Materials (Basel). 2021 May 29;14(11):2930. doi: 10.3390/ma14112930 (PMC8199491; doi:10.3390/ma14112930)
Supplement: Supplementary file 1 [file materials-14-02930-s001.zip › materials-1224800-SI.pdf]

Supplementary Materials

Composite Fe_3O_4-MXene-Carbon Nanotube Electrodes for Supercapacitors Prepared Using the New Colloidal Method

Wenyu Liang and Igor Zhitomirsky *

Department of Materials Science and Engineering, McMaster University, Hamilton, ON L8S4L7, Canada; liangw26@mcmaster.ca

* Correspondence: zhitom@mcmaster.ca

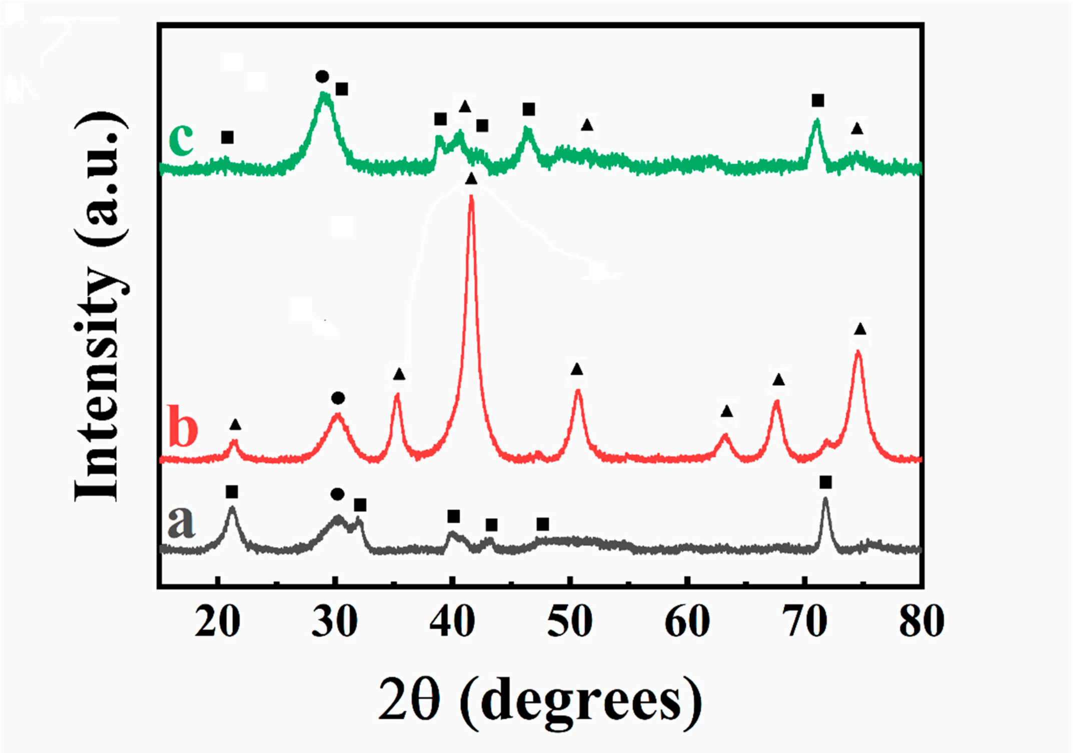

Figure S1. X-ray diffraction patterns of (**a**) $Ti_3C_2T_x$-CNT and (**b**) Fe_3O_4-CNT and (**c**) $Ti_3C_2T_x$-Fe_3O_4-CNT composites (■ - $Ti_3C_2T_x$, ● – CNT, ▲ – Fe_3O_4).

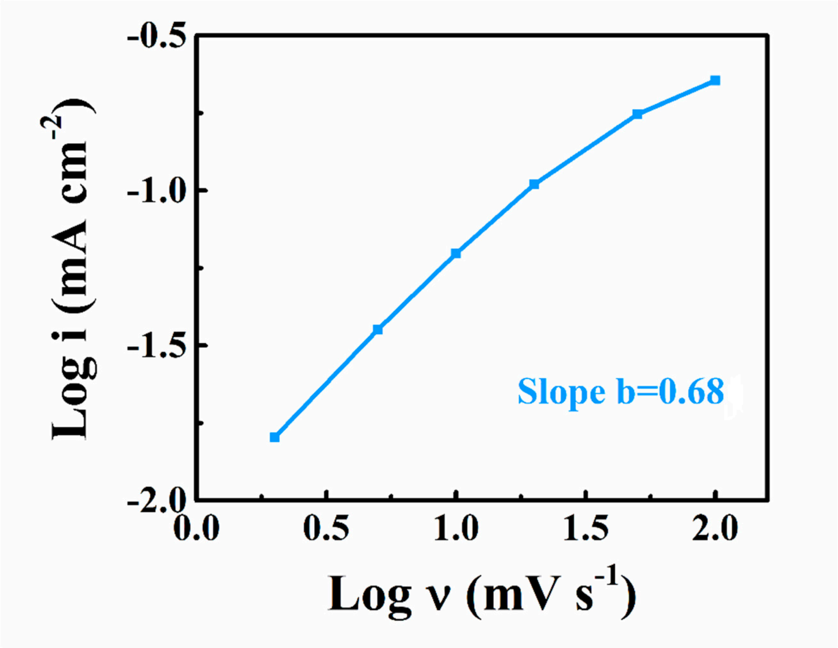

Figure S2. Current (i) versus scan rate (ν) dependence in a logarithmic scale used for the calculation of parameter b for $Ti_3C_2T_x$-Fe_3O_4-CNT electrodes from the equation [1] $i = av^b$.

Table S1. Characteristics of $Ti_3C_2T_x$-based electrodes with high active mass in Na_2SO_4 electrolyte.

Material	Active Mass mg·cm^{-2}	Potential Window	Areal Capacitance F·cm^{-2}	Reference
$Ti_3C_2T_x$-acetylene black	20	0–0.9V*	1.087	[2]
$Ti_3C_2T_x$-CNT	35	−1.1–−0.3 V vs SCE	1.93	[3]
$Ti_3C_2T_x$-CNT	40	−1.1–−0.3 V vs SCE	2.26	[4]
$Ti_3C_2T_x$-Fe_3O_4-CNT	35	−1.1–−0.3 V vs SCE	5.52	This work

•Measurements performed in two electrode configuration

Reference

1. Okhay, O.; Tkach, A.; Graphene/Reduced Graphene Oxide-Carbon Nanotubes Composite Electrodes: From Capacitive to Battery-Type Behaviour. *Nanomaterials* **2021**, *11*, 1240.
2. Guo, M.; Liu, C.; Zhang, Z.; Zhou, J.; Tang, Y.; Luo, S. Flexible Ti3C2Tx@ Al electrodes with ultrahigh areal capacitance: in situ regulation of interlayer conductivity and spacing. *Adv. Funct. Mater.* **2018**, *28*, 1803196.
3. Liang, W.; Zhitomirsky, I.; MXene–carbon nanotube composite electrodes for high active mass asymmetric supercapacitors. *J. Mater. Chem.* A **2021**, *9*, 10335–10344.
4. Li, X.; Zhu, J.; Liang, W.; Zhitomirsky, I.; MXene (Ti3C2Tx) Anodes for Asymmetric Supercapacitors with High Active Mass Loading. *Mater. Chem. Phys.* **2021**, in press.
